# Robotic Versus Sternotomy Approach for Left Atrial Myxoma Resection: A Retrospective Single-Center Study

**DOI:** 10.3390/jcm14228220

**Published:** 2025-11-20

**Authors:** Gabriel Saiydoun, Saadé Saade, Costin Radu, Eric Bergoend, Thierry Folliguet

**Affiliations:** 1 Department of Cardiac and Thoracic Surgery, Pitié-Salpêtrière University Hospital, Assistance Publique des Hôpitaux de Paris, 75013 Paris, France; 2 Department of Cardiac Surgery, Strasbourg University Hospital, 67091 Strasbourg, France; 3 Department of Cardiac and Thoracic Surgery, Henri Mondor University Hospital, Assistance Publique des Hôpitaux de Paris, 94000 Créteil, France

**Keywords:** robotic cardiac surgery, minimally invasive surgery, cardiac tumor, innovation in cardiac surgery

## Abstract

**Objectives**: This study aimed to compare survival and outcomes between robotic-assisted and conventional sternotomy myxoma resection. **Methods**: This retrospective single-center study included 16 consecutive patients undergoing left atrial myxoma resection between April 2019 and June 2024. All procedures were performed by the same surgical team. The robotic approach involved peripheral cardiopulmonary bypass (CPB), Custodiol^®^ cardioplegia, and DaVinci Xi^®^ via right mini-thoracotomy. The primary endpoint was 30-day cerebrovascular accident-free survival. Secondary outcomes included 5-year survival, stroke, pacemaker implantation, bleeding, Intensive care unit, and hospital stay. **Results**: Sixteen patients were included (8 robotic, 8 sternotomy); median age was 58.0 [IQR 53.2–67.8] in the robotic group and 66.6 [62.0–71.0] years in the sternotomy group, with a similar sex distribution between groups. No significant baseline differences between groups except a lower EuroSCORE II in the robotic group (0.8% vs. 1.3%, *p* = 0.004). Robotic surgery resulted in significantly longer CPB time (181 vs. 46 min, *p* < 0.001) and cross-clamp time (67 vs. 31 min, *p* < 0.001), but similar intensive care unit stay (2.5 vs. 2.6 days, *p* = 0.95) and hospital stay (8.5 vs. 8.4 days, *p* = 0.87). At 30 days, stroke-free survival was 100% in both groups (*p* > 0.9). At 5 years, survival remained 100% in the robotic group versus 86% in the sternotomy group (*p* = 0.47). No conversions, reinterventions, or major postoperative complications were observed. **Conclusions**: Robotic-assisted resection of left atrial myxomas appears to be feasible and safe in a selected low-risk cohort, when compared with conventional sternotomy, with excellent mid-term survival despite longer operative times.

## 1. Introduction

In France, the uptake of robotic cardiac surgery has been modest, primarily due to a lack of specific regulatory approvals, limited reimbursement options, and operational challenges within surgical teams. The da Vinci^®^ system is not specifically authorized or reimbursed for cardiac procedures, restricting its use to research or self-funded programs. Concerns also persist about prolonged cross-clamp and bypass times, which require rapid conversion capabilities not available in all centers. Although robotic cardiac surgery has gained ground in various European countries, France has yet to develop a network of high-volume institutions equipped with formalized training and consistent procedural support [1]. As a result, robotic procedures remain confined to a few expert teams. Broader adoption will require national investment in training infrastructure, formal approval of cardiac indications, and large-scale clinical validation. This evolution reflects a broader trend toward minimizing surgical trauma, reducing hospital stay, and enhancing cosmetic outcomes, while maintaining procedural safety and effectiveness. Despite these advances, concerns remain regarding the increased cardiopulmonary bypass and aortic cross-clamp times often associated with robotic techniques, particularly in complex intracardiac procedures. Nevertheless, evidence continues to accumulate in support of robotic cardiac surgery as a viable and safe alternative to conventional approaches [2]. In particular, robotic-assisted surgery for left atrial myxoma resection has been described in several series as technically feasible, with low morbidity, excellent visualization of the interatrial septum, and promising long-term outcomes [3,4,5].

Primary cardiac tumors are rare, with an incidence of approximately 0.001% to 0.03% in autopsy series, and myxomas represent the most common benign subtype, typically originating in the left atrium. Given their embolic potential and obstructive risk, surgical resection remains the standard of care. While median sternotomy has long been the reference approach, robotic-assisted myxoma resection has emerged as an attractive minimally invasive option in selected patients [4,5].

The objective of this investigation was to assess and contrast mid-term survival rates, safety profiles, and perioperative metrics associated with robotic-assisted versus traditional sternotomy-based resection of left atrial myxomas. By analyzing a consecutive single-center cohort with mid-term follow-up, we sought to evaluate whether the robotic technique offers equivalent or superior clinical results, while reducing surgical invasiveness and enhancing postoperative recovery.

## 2. Materials and Methods

### 2.1. Study Population

This retrospective study included 16 consecutive adult patients who underwent surgical resection of a left atrial myxoma at Créteil University Hospital between April 2019 and June 2024. Patients were divided into two groups according to the surgical approach: robotic-assisted (*n* = 8) and conventional median sternotomy (*n* = 8). All procedures were performed by the same surgical team. Selection of the patients for minimally invasive resection included adult patients (>18 years) presenting with a single left atrial myxoma confirmed by echocardiography and undergoing isolated myxoma resection. Exclusion criteria included the presence of concomitant cardiac pathologies requiring associated procedures (such as coronary artery bypass grafting, aortic, mitral or tricuspid valve surgery), prior cardiac surgery, active endocarditis, or significant pulmonary disease contraindicating single-lung ventilation. The echocardiogram was performed and read by two in-hospital cardiologists and after discharge, all echocardiograms were performed both by the referring cardiologist and by one of the two in-hospital cardiologists.

### 2.2. Study Approval

This study was approved by the Institutional Review Board (IRB00012919) of the French Society of Thoracic and Cardiovascular Surgery (CERC-SFCTCV-2025–10-21_40728). All patients provided written informed consent for the use of their anonymized medical data for research purposes. The data underlying this article will be shared on reasonable request to the corresponding author.

### 2.3. Robotic Surgical Technique

In the robotic-assisted group, all procedures were performed using the Da Vinci Xi^®^ surgical robotic systems (Intuitive, Sunnyvale, CA, USA) through a right mini-thoracotomy approach.

### 2.4. Preparation

Patients were positioned supine with a slight lateral tilt to the right, the right arm tucked alongside the body, mimicking the setup typically used for right-sided thoracotomy. A double-lumen endotracheal tube was inserted with right lung deflation. Selective left lung ventilation was achieved via double-lumen endotracheal intubation to optimize exposure of the left atrium. A left radial arterial catheter was placed, and external defibrillator patches were placed on the thoracic cage.

### 2.5. Exposition

Cardiopulmonary bypass was established peripherally through femoral artery and vein cannulation. After systemic heparinization the right femoral vessels were surgically cannulated with a 27F or 29F cannula in the vein, and an 18F cannula in the artery. Additionally, a 17F wirebound cannula was inserted in the right internal jugular vein for optimal drainage. Cardiopulmonary bypass was instituted and cooling to 33 °C rectal temperature was obtained. A 4–5 cm lateral incision was made in the fourth intercostal space. A 30-degree angled camera was placed in the same intercostals space anterior. Two ports were placed, one in the 2nd or 3rd intercostal space mid axillary and one in the 6 or seventh intercostal. Three robotic ports were inserted in the right chest wall: 2 for working arms and one for the camera, along with an auxiliary retraction port. This setup provided excellent access to the interatrial septum and allowed precise tumor excision under high-definition 3D visualization.

Continuous CO_2_ irrigation was maintained in the pleural cavity thought a separate port until aortic declamping was performed. The heart was arrested with cold custodiol cardioplegia in the aortic root. Myocardial protection was ensured using single-dose cold crystalloid cardioplegia (Custodiol^®^ solution). The ascending aorta was cross-clamped with a transthoracic aortic cross clamp placed either through the second intercostal space mid axillary’s line, or directly placed via the working port. Soft tissue retractors were used, and no rib spreading were used.

### 2.6. Operation

The pericardium was opened from the inferior above the diaphragm and extended toward the superior vena cava. The left atrium was opened longitudinally, and a left atrial retractor was inserted (Heart port, Inc., Redwood City, CA, USA).

The left atrium was inspected and a 4-0 Gore-Tex stitch was placed at the inferior part of the atrial incision to allow for traction.

The myxoma was resected with its base using cautery scissors. When needed the septum was closed with a 4-0 Prolene. The atrial incision was closed with the 4 0 Goretex already placed moving superiorly and a second 4-0 Goretex suture was used starting at the top of the incision moving down toward the other sutures and ending in the middle of the incision.

### 2.7. Completion

Deairing was performed in the following sequence, we aspirated on both the aortic cardioplegia line as well as through a left atrial line while both lung ventilation was performed, and the heart was being filled. At the same time continuous CO_2_ irrigation was being maintained, after ventilation had been resumed, we then clamped the left atrial line and aspirated only on the ascending aorta line. We monitored the presence of air on the TEE and declamped the aorta after visualizing the absence of air in the left heart cavities. Cardiopulmonary bypass was then weaned and the cardioplegia line was removed and secured with two sutures.

### 2.8. Follow-Up and Study Endpoints

Demographic, perioperative, and postoperative data were collected from medical records. The primary outcome was 30-day cerebrovascular accident (CVA)-free survival. Secondary outcomes included overall 5-year survival, postoperative stroke, permanent pacemaker (PM) implantation, bleeding, length of ICU stay, and total hospital stay.

Postoperative events were defined a priori using standardized clinical and radiological criteria:

− Stroke: new focal neurological deficit lasting ≥ 24 h and confirmed by cerebral imaging.
− Bleeding: transfusion of >2 units of packed red cells within 24 h or re-exploration for bleeding.
− Acute kidney injury (AKI): AKIN stage ≥ 3 (serum creatinine ≥ 3 × baseline or new dialysis requirement).
− Pneumonia: new radiographic infiltrate associated with fever > 38 °C, leukocytosis or leukopenia, and positive sputum or bronchoalveolar culture.
− Wound or port-site infection: erythema, drainage, or collection requiring antibiotic therapy or surgical revision.
− Tamponade: postoperative pericardial effusion requiring surgical evacuation.
− Transfusion: administration of any allogeneic red blood cell unit postoperatively.

### 2.9. Statistical Analysis

Continuous variables are expressed as medians with interquartile ranges (IQR) and 95% confidence intervals (CIs). Categorical variables are presented as proportions with exact Clopper–Pearson 95% CIs, and between-group risk differences were computed with 95% CIs according to the Newcombe method.

For clarity, medians and IQRs are displayed in the main tables, whereas the corresponding 95% CIs and between-group differences are provided in the Supplementary Material (Supplementary Tables S1–S3).

Comparative statistical analyses between the two groups were performed using Mann–Whitney U tests for continuous variables and Fisher’s exact tests for categorical variables. In view of the small cohort size, multivariable modeling was limited to a simple binary logistic regression including EuroSCORE II (continuous) as covariate, with the dependent variable defined as any postoperative complication (death, stroke, reoperation, tamponade, pneumonia, dialysis, or transfusion). The model was intended to evaluate whether the difference in baseline operative risk affected outcomes between surgical approaches. Survival at one and five years was verified using follow-up data and national vital statistics.

Because all patients had complete follow-up and no censoring occurred, survival is reported as observed proportions rather than Kaplan–Meier estimates. No missing data were present for any variable. A *p*-value of <0.05 was considered statistically significant. All statistical analyses were performed using R statistical software (version 4.1.1; R Core Team, 2021).

## 3. Results

### 3.1. Study Population

A total of 16 consecutive patients who underwent left atrial myxoma resection between 2019 and 2024 were included, equally divided into robotic-assisted (*n* = 8) and sternotomy (*n* = 8) groups. Baseline demographic and preoperative characteristics are summarized in Table 1. There were no significant differences between groups regarding age, comorbidities, or cardiac history. Median age was lower in the robotic group (58.0 years [IQR 53.2–67.8]) compared to the sternotomy group (66.6 years [IQR 62.0–71.0]; *p* = 0.312). The EuroSCORE II was significantly lower in the robotic group (0.8% vs. 1.3%, *p* = 0.004), reflecting a lower estimated surgical risk.

The median follow-up was 58 months [IQR 42–67], and no patient was lost to follow-up.

No patient had prior cardiac surgery, and functional class distribution (NYHA) and LVEF were comparable between the two groups. There were no statistically significant differences in renal, respiratory, or metabolic comorbidities.

### 3.2. Survival Outcomes

**Figure 1:**
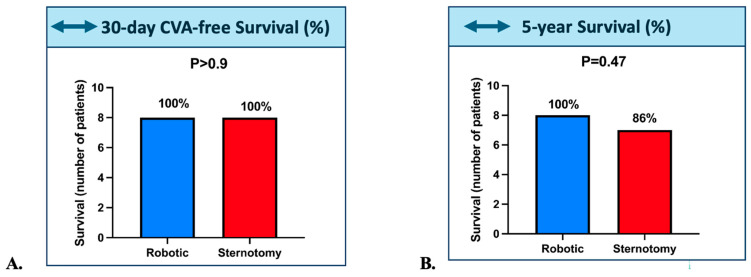
(**A**). 30-day Stroke-Free Survival and (**B**). 5-year survival in the Robotic (Blue) and Sternotomy (Red) groups; Abbreviations: CVA: cerebrovascular accident. The horizontal arrow indicates the comparison between groups, showing no significant difference.

At 30 days, stroke-free survival was 100% in both groups (Figure 1A, *p* > 0.9). Long-term outcomes were similarly favorable. At 5 years, survival remained 100% in the robotic group and was 86% in the sternotomy group (1 death), without reaching statistical significance (Figure 1B, *p* = 0.47).

No patient in the robotic group required conversion to sternotomy. Throughout the follow-up period, no reinterventions, readmissions, or cardiovascular events were observed, reinforcing the safety and durability of both approaches.

### 3.3. Intraoperative and Postoperative Outcomes

**Figure 2:**
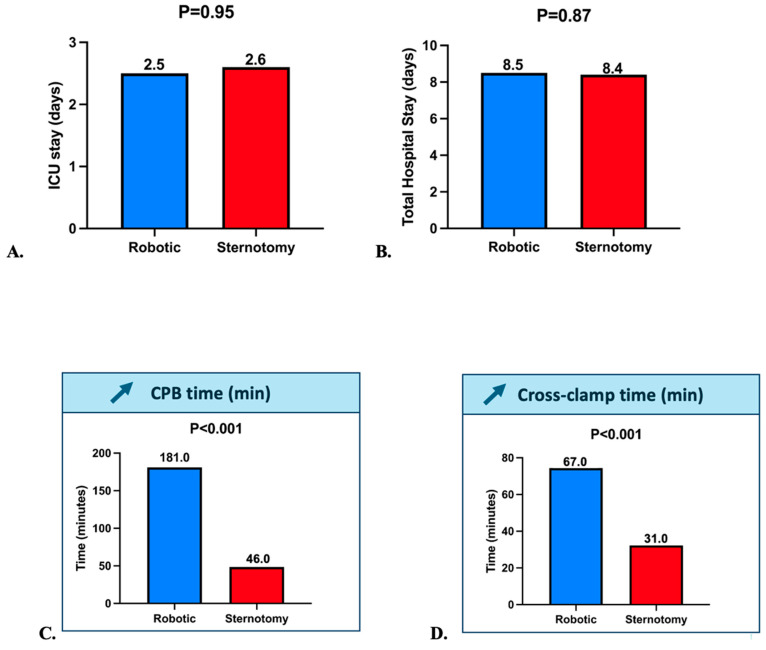
(**A**). ICU Stay in the Robotic (Blue) and Sternotomy (Red) groups (**B**). Total Hospital Stay in the Robotic (Blue) and Sternotomy (Red) groups (**C**). CPB time in the Robotic (Blue) and Sternotomy (Red) groups (**D**). Cross-Clamp time in the Robotic (Blue) and Sternotomy (Red) groups. Abbreviations: CPB: Cardio-pulmonary bypass; ICU: Intensive care unit. The upward arrow indicates a significant increase in the robotic group compared with the sternotomy group.

Operative and perioperative outcomes are detailed in Table 2. There was no significant difference in ICU stay (2.5 vs. 2.6 days, *p* = 0.95) or total hospital stay (8.5 vs. 8.4 days, *p* = 0.87), as shown in Figure 2A,B. Robotic procedures were associated with significantly longer cardiopulmonary bypass times (181.0 vs. 46.0 min, *p* < 0.001) and cross-clamp times (67.0 vs. 31.0 min, *p* < 0.001), as illustrated in Figure 2C,D. Despite these longer operative durations, postoperative recovery was comparable.

No cases of bleeding, tamponade, reoperation, stroke, or pacemaker implantation were observed in either group. Only one case of postoperative acute renal failure (AKIN 3) occurred in the sternotomy group. Two patients in the robotic group had a diagnosis of lymphoma. No postoperative transfusions, pneumonia, or urinary tract infections were reported in either group.

### 3.4. Sensitivity Analysis

After adjustment for EuroSCORE II in a binary logistic regression model, the surgical approach (robotic vs. sternotomy) was not significantly associated with postoperative complications (adjusted OR = 0.94; 95% CI 0.12–7.65; *p* = 0.96). This finding suggests that the lower baseline EuroSCORE II in the robotic group did not confound the absence of differences in observed outcomes.

## 4. Discussion

### 4.1. Summary of Findings

In this single-center retrospective study comparing robotic-assisted and conventional sternotomy approaches for left atrial myxoma resection, both groups achieved 100% 30-day stroke-free survival, with no mortality, conversion, or need for pacemaker implantation. Complete tumor excision was accomplished in all patients, with no recurrence during mid-term follow-up. Notably, cardiopulmonary bypass and aortic cross-clamp times were significantly longer in the robotic group. Despite these prolonged durations, the robotic approach did not result in higher complication rates or longer ICU and hospital stays.

### 4.2. Comparison with Existing Literature

Robotic-assisted cardiac surgery has progressively emerged as a viable alternative to conventional sternotomy for selected cardiac procedures, including the resection of benign cardiac tumors such as left atrial myxomas. According to Cerny et al., robotic cardiac surgery remains underdeveloped in several European countries, with only a handful of high-volume centers routinely performing these procedures [1]. Despite the lack of widespread adoption, outcomes remain favorable in experienced teams. Bush et al. provided historical context, highlighting the evolution of robotic cardiac surgery and emphasizing its growing role in minimally invasive intracardiac procedures, where safety and precision are critical [2].

In line with our findings, prior case series have confirmed the safety and feasibility of robotic myxoma resection. In their series, Schilling et al. highlighted the safety and technical feasibility of robotic atrial myxoma excision, emphasizing the superior visualization of the interatrial septum afforded by the robotic platform [3]. Kesävuori et al. further showed that patients undergoing robotic excision of atrial myxomas reported high postoperative quality-of-life scores, which is particularly relevant in the context of benign tumors managed electively [4]. Gao et al. also confirmed the safety and reproducibility of the robotic approach in a series of patients with atrial myxoma, noting improved exposure and reduced trauma to the chest wall [5].

Large-scale data support the notion that outcomes in robotic cardiac surgery are highly volume-dependent. In a national cohort of over 10,000 robotic cardiac procedures, Zhuli et al. demonstrated that hospital volume was strongly associated with reduced complications and improved outcomes, suggesting that institutional experience remains a key determinant of success [6]. Wah echoed this observation, noting that robotic cardiac surgery yields the most benefit in settings where training, team coordination, and perioperative protocols are optimized through experience and repetition [7]. These findings align with our center’s results, where all robotic procedures were performed by a dedicated and experienced team, minimizing variability and supporting the reproducibility of the technique.

The lower EuroSCORE II in the robotic group resulted from deliberate early-adoption selection of lower-risk cases and logistical factors (availability of the robotic platform/team) that diverted otherwise eligible cases to sternotomy on certain days. Conversely, patients with higher EuroSCORE II, advanced age, or significant comorbidities were preferentially treated via sternotomy as a safety measure.

Beyond safety, quality-of-life benefits have been a central argument for robotic approaches. As demonstrated by Currie et al., robotic-assisted coronary revascularization was associated with superior patient-reported outcomes, including earlier return to work and reduced postoperative discomfort [8]. These advantages were also noted in robotic tumor resection: Deshpande et al. reported high satisfaction scores among patients undergoing totally endoscopic excision of intracardiac tumors [9]. Similarly, Gao et al. and Hassan et al. emphasized the cosmetic and recovery-related benefits of robotic access, particularly in younger or low-risk patients [10,11].

The robotic approach to benign tumor resection is not without technical challenges, particularly regarding operative times. Our study confirms that cardiopulmonary bypass and cross-clamp durations are longer in robotic procedures, a finding echoed by Murphy et al. in their early robotic series [12]. Nonetheless, these extended times did not result in worse outcomes, consistent with the experience of Nifong et al., who managed 540 robotic mitral cases with low morbidity despite prolonged bypass durations in early cases [13]. Schilling et al. also emphasized that surgical exposure and tumor resection can be safely achieved robotically, provided meticulous preoperative planning and a standardized intraoperative strategy [3].

Robotic cardiac surgery has also been associated with improved functional outcomes and long-term quality of life. Bonaros et al. showed that patients undergoing robotic coronary artery bypass grafting reported sustained improvements in physical and emotional well-being [14]. Similar findings were reported by Morgan et al. in patients undergoing robotic ASD repair [15], and by Suri et al., who compared robotic and conventional mitral valve repairs, showing better short-term recovery profiles in the robotic group [16]. These results support the notion that robotic approaches may offer long-term value beyond immediate perioperative outcomes, particularly for benign conditions like myxoma.

Finally, the evidence base supporting robotic myxoma surgery continues to grow. Russo et al. compared minimally invasive and standard approaches for excising atrial masses and reported shorter recovery times and similar complication rates for the minimally invasive cohort [17]. In Finland, Kesävuori et al. validated the quality-of-life gains after robotic tumor excision using standard instruments such as the RAND-36 questionnaire [4]. Collectively, these findings suggest that, in selected patients and expert hands, robotic surgery for atrial myxoma provides an attractive alternative to sternotomy, balancing safety, efficacy, and patient-centered outcomes [18,19].

In addition to tumor resection, robotic and totally endoscopic approaches have also been successfully applied to coronary surgery, including in high-risk and reoperative settings. Yamashita et al. recently demonstrated the feasibility and safety of robotic-assisted redo coronary artery bypass grafting, even in a cohort with elevated STS scores and complex revascularization strategies, with no conversions or mortality [20]. In a nine-month single-center start-up, Piperata et al. reported 29 consecutive robotic mitral repairs using a respect-not-resect strategy with neochordae and annuloplasty. Procedural success was 97% with 0% 30-day cardiac mortality; median ICU and hospital stays were 1 and 8 days, respectively.

Median CPB and cross-clamp times were 189 and 111 min, decreasing progressively with experience [21]. Careful patient selection, a dedicated team, and robust prior mitral repair expertise enabled a rapid learning curve despite no prior minimally invasive program. Similarly, Goto et al. described a standardized, efficient, and reproducible method for totally endoscopic internal thoracic artery harvesting in minimally invasive coronary bypass surgery, achieving universal graft patency without complications or sternotomy conversion [22]. These findings highlight the growing versatility of robotic platforms beyond valvular and tumor surgery, reinforcing their potential for broader integration into complex cardiac procedures. Despite the growing evidence supporting minimally invasive and robotic cardiac surgery, the learning curve and safety of wider implementation remain key concerns. Proper team training, case selection, and conversion readiness are essential to maintain outcomes comparable to open surgery. Recent randomized data from the UK Mini Mitral Trial demonstrated that minithoracotomy was not superior to sternotomy for degenerative mitral valve repair in terms of functional recovery or safety outcomes at 1 year, underscoring the need for structured adoption and training when extending minimally invasive programs [23].

### 4.3. Limitations

This study has several limitations. First, the small sample size, inherent to the rarity of left atrial myxomas, limits the statistical power and generalizability of our findings. Second, the retrospective design and lack of randomization introduce potential selection bias, as patients in the robotic group had a slightly lower EuroSCORE II. Third, although logistic regression adjustment was performed, the limited sample size may affect the robustness of the adjusted analyses. Finally, all procedures were performed by a single experienced surgical team, which may not reflect broader practice settings.

## 5. Conclusions

The robotic approach for left atrial myxoma resection demonstrated feasibility and safety in a selected low-risk cohort, when compared with conventional sternotomy, despite significantly longer cardiopulmonary bypass and cross-clamp times. It was associated with similar recovery durations, no increase in perioperative morbidity, and excellent five-year survival. Notably, no conversions to sternotomy were required, underscoring the feasibility and reliability of the robotic technique in experienced centers. Our findings contribute to the growing body of evidence favoring robotic strategies for cardiac tumor removal and suggest a compelling case for further integration of minimally invasive technologies in cardiac surgical practice.

## Figures and Tables

**Table 1:** Baseline Demographic and Preoperative Characteristics of Patients Undergoing Left Atrial Myxoma Resection via Robotic versus Sternotomy Approach.

Variable	Sternotomy (Median [IQR])	Robotic (Median [IQR])	*p*-Value
Age	66.6 [62.0–71.0]	58.0 [53.2–67.8]	0.312
Weight (kg)	78.5 [62.5–88.0]	66.3 [63.0–75.0]	0.289
Height (cm)	165.5 [158.8–170.5]	165.5 [161.5–171.2]	0.875
Hypertension	0.0 [0.0–1.0]	0.0 [0.0–0.5]	0.777
Dyslipidemia	0.0 [0.0–0.2]	0.0 [0.0–0.5]	0.94
Tobacco use	0.0 [0.0–0.0]	0.0 [0.0–0.0]	0.54
NYHA			0.2
I	1	4	
II	3	0	
III	2	4	
IV	2	0	
LVEF (%)	60.0 [60.0–64.2]	63.0 [59.0–66.0]	0.789
sPAP (mmHg)	25.0 [25.0–26.5]	24.0 [23.0–25.0]	0.335
Prior PM	0.0 [0.0–0.0]	0.0 [0.0–0.0]	1
Prior Cardiac Surgery	0/8 (0.0%)	0/8 (0.0%)	1
PAD	0/8 (0.0%)	0/8 (0.0%)	1
Prior Stroke	0/8 (0.0%)	1/7 (14.3%)	0.467
Diabetes	1/8 (12.5%)	1/8 (12.5%)	1
Chronic renal failure	2/8 (25.0%)	0/8 (0.0%)	0.467
Dialysis	1/8 (12.5%)	0/8 (0.0%)	1
COPD	1/8 (12.5%)	1/8 (12.5%)	1
Endocaditis	0/8 (0.0%)	0/8 (0.0%)	1
History of MI	0/8 (0.0%)	0/8 (0.0%)	1
Prior Anticoagulation	1/8 (12.5%)	0/8 (0.0%)	1
Prior Antiaggregation	4/8 (50.0%)	2/7 (28.6%)	0.608
EuroSCORE II (%)	1.3 [1.0–2.4]	0.8 [0.6–0.9]	0.004
CPB Time (min)	46.0 [34.0–54.0]	181.0 [169.0–206.0]	0.001
Cross-clamp time (min)	31.0 [24.0–39.0]	67.0 [64.0–74.4]	0.001

CPB: Cardiopulmonary Bypass COPD: Chronic Obstructive Pulmonary Disease; LVEF: Left Ventricular Ejection Fraction; MI: Myocardial Infarction; NYHA: New York Heart Association; sPAP Systolic Pulmonary Artery Pressure; PM: Pacemaker; PAD: Peripheral Artery Disease.

**Table 2:** Outcomes of Patients Undergoing Left Atrial Myxoma Resection via Robotic versus Sternotomy Approach.

Variable	Sternotomy (Median [IQR])	Robotic (Median [IQR])	*p*-Value
5-Year Death	1/8 (12.5%)	0/8 (0.0%)	1
Bleeding	0/8 (0.0%)	0/8 (0.0%)	1
Tamponnade	0/8 (0.0%)	0/8 (0.0%)	1
Operative site Infection	0/8 (0.0%)	0/8 (0.0%)	1
Reoperation	0/8 (0.0%)	0/8 (0.0%)	1
Postoperative MI	0/8 (0.0%)	0/8 (0.0%)	1
Post-operative PM	0/8 (0.0%)	0/8 (0.0%)	1
Post-operative stroke	0/8 (0.0%)	0/8 (0.0%)	1
Renal failure (AKIN 3)	1/8 (12.5%)	0/8 (0.0%)	1
ARDS	0/8 (0.0%)	0/8 (0.0%)	1
Pneumonia	0/8 (0.0%)	0/8 (0.0%)	1
Postop Transfusion	0.0 [0.0–0.0]	0.0 [0.0–0.0]	1
UTI	0.0 [0.0–0.0]	0.0 [0.0–0.0]	1

AKIN: Acute Kidney Injury Network; ARDS: Acute Respiratory Distress Syndrome; MI: Myocardial Infarction; PM: Pacemaker; UTI: Urinary Tract Infection.

## Data Availability

Data are available after formal request to and acceptance by the Ethical Committee of Clinical Research of the French Society of Thoracic and Cardiovascular Surgery (comite-ethique@sfctcv.org).

## Supplementary Materials

The following supporting information can be downloaded at: https://www.mdpi.com/article/10.3390/jcm14228220/s1, Table S1: 95% Confidence Intervals for Continuous Variables; Table S2: Median Differences (Robotic–Sternotomy) with 95% Cis; Table S3: Standardized Mean Differences (SMDs) for All Variables; Video S1: Totally endoscopic robotic-assisted resection of a left atrial myxoma using the Da Vinci Xi^®^ surgical robotic systems (Intuitive, Sunnyvale, CA, USA) through a right mini-thoracotomy.

## Abbreviations

AKIN	Acute Kidney Injury Network
ARDS	Acute Respiratory Distress Syndrome
CPB	Cardiopulmonary Bypass
COPD	Chronic Obstructive Pulmonary Disease
CVA	Cerebrovascular Accident
HTK (Custodiol^®^)	Histidine-Tryptophan-Ketoglutarate cardioplegic solution
ICU	Intensive Care Unit
IQR	Interquartile Range
LVEF	Left Ventricular Ejection Fraction
MI	Myocardial Infarction
NYHA	New York Heart Association (functional classification)
PAD	Peripheral Artery Disease
PM	Pacemaker
sPAP	Systolic Pulmonary Artery Pressure
TEE	Transesophageal Echocardiography
