# Supplementary material for: Robotic Versus Sternotomy Approach for Left Atrial Myxoma Resection: A Retrospective Single-Center Study
Source: J Clin Med. 2025 Nov 20;14(22):8220. doi: 10.3390/jcm14228220 (PMC12653283; doi:10.3390/jcm14228220)
Supplement: Supplementary file 1 [file jcm-14-08220-s001.zip › jcm-3894178-supplementary.pdf]

Supplementary Table S1. 95% Confidence Intervals for Continuous Variables

Variable	95% CI (Sternotomy)	95% CI (Robotic)
Age	[58.3–70.4]	[49.2–66.1]
Weight (kg)	[63.1–85.9]	[58.2–73.4]
Height (cm)	[158.3–170.1]	[159.2–171.7]
LVEF (%)	[59–64]	[58–68]
sPAP (mmHg)	[24.5–26.8]	[22.9–25.3]
EuroSCORE II (%)	[1.00–3.95]	[0.61–0.92]
CPB Time (min)	[38.1–51.9]	[173.2–200.8]
Cross-clamp Time (min)	[27.0–37.5]	[65.0–72.3]

CI: Confidence Interval, LVEF: Left Ventricular Ejection Fraction, sPAP: Systolic Pulmonary Artery Pressure, EuroSCORE II: European System for Cardiac Operative Risk Evaluation II, CPB Time: Cardiopulmonary Bypass Time.

Supplementary Table S2. Median Differences (Robotic – Sternotomy) with 95% CIs

Variable	Median Difference (R–S)	95% CI of Difference
Age	−8.6	[−21.4 to 5.3]
Weight (kg)	−12.2	[−28.4 to 4.1]
Height (cm)	0.0	[−6.5 to 6.3]
LVEF (%)	+3	[−2 to 8]
sPAP (mmHg)	−1.0	[−2.4 to 0.6]
EuroSCORE II (%)	−0.52	[−1.10 to −0.04]
CPB Time (min)	+135	[+124 to +145]
Cross-clamp Time (min)	+36	[+32 to +40]
Hypertension	−12.5	[−47.3 to 22.3]
Dyslipidemia	0.0	[−32.4 to 32.4]
Tobacco use	0.0	[−32.4 to 32.4]
Diabetes	0.0	[−32.4 to 32.4]
Chronic renal failure	−25	[−58 to 15]
COPD	0.0	[−32.4 to 32.4]
Prior Stroke	+14.3	[−13.5 to 44.6]
PAD	0.0	[−32.4 to 32.4]
Prior PM	0.0	[−32.4 to 32.4]
Prior Cardiac Surgery	0.0	[−32.4 to 32.4]
Endocarditis	0.0	[−32.4 to 32.4]
History of MI	0.0	[−32.4 to 32.4]
Prior Anticoagulation	−12.5	[−47.1 to 21.5]
Prior Antiaggregation	−21.4	[−58.2 to 11.9]

CI: Confidence Interval, LVEF: Left Ventricular Ejection Fraction, sPAP: Systolic Pulmonary Artery Pressure, EuroSCORE II: European System for Cardiac Operative Risk Evaluation II, CPB Time: Cardiopulmonary Bypass Time, COPD: Chronic Obstructive Pulmonary Disease, PAD: Peripheral Arterial Disease, PM: Pacemaker, MI: Myocardial Infarction.

Supplementary Table S3 – Standardized Mean Differences (SMDs) for All Variables

Variable	SMD
Age	0.346
Weight (kg)	0.289
Height (cm)	0.041
LVEF (%)	0.257
sPAP (mmHg)	0.176
EuroSCORE II (%)	0.512
CPB Time (min)	1.922
Cross-clamp Time (min)	1.853
Hypertension	0.256
Dyslipidemia	0.000
Tobacco use	0.000
Diabetes	0.000
Chronic renal failure	0.251
COPD	0.000
Prior Stroke	0.223
PAD	0.000
Prior PM	0.000
Prior Cardiac Surgery	0.000
Endocarditis	0.000
History of MI	0.000
Prior Anticoagulation	0.196
Prior Antiaggregation	0.326

SMD: Standardized Mean Difference, LVEF: Left Ventricular Ejection Fraction, sPAP: Systolic Pulmonary Artery Pressure, EuroSCORE II: European System for Cardiac Operative Risk Evaluation II, CPB Time: Cardiopulmonary Bypass Time, COPD: Chronic Obstructive Pulmonary Disease, PAD: Peripheral Arterial Disease, PM: Pacemaker, MI: Myocardial Infarction.
